# Bioprospecting cultivable bacteria associated with deep sea (mesopelagic) fish of the North Atlantic Ocean

**DOI:** 10.1007/s13659-025-00527-6

**Published:** 2025-07-02

**Authors:** Ernest Oppong-Danquah, Jana Heumann, Hannah Moosbauer, Martina Blümel, Arlette Wenzel-Storjohann, Deniz Tasdemir

**Affiliations:** 1 GEOMAR Centre for Marine Biotechnology (GEOMAR-Biotech), Research Unit Marine Natural Products Chemistry, GEOMAR Helmholtz Centre for Ocean Research Kiel, Kiel, Germany; 2 Kiel University, Kiel, Germany

**Keywords:** Mesopelagic zone, Fish, Bioprospecting, Antibiotic activity, Untargeted metabolomics, Feature-based molecular networking

## Abstract

The microbiota associated with fish is increasingly recognized as a valuable source of bioactive metabolites for pharmaceutical application. The mesopelagic zone, a deep and unique ecosystem with a diverse biological community, is among the least studied marine environments. This study explored the potential of cultivable microbiota associated mainly with mesopelagic fish for pharmaceutical and agricultural applications. We isolated and identified 643 cultivable bacteria predominantly from various organs of fish collected from the mesopelagic zone of the North Atlantic Ocean, with additional samples from jellyfish, squid and krill. The bacterial community was dominated by the Gram-negative phylum Pseudomonadota, particularly the genera *Psychrobacter*, *Pseudoalteromonas* and *Vibrio*. A total of 394 bacterial isolates were selected and cultured in two growth media. Microbial extracts (590) were assessed for their anticancer and antimicrobial activities against human and fish pathogens. Over 60% of extracts exhibited activity against two ESKAPE pathogens methicillin-resistant *Staphylococcus aureus* (MRSA) and *Enterococcus faecium*, as well as the fish pathogen *Lactococcus garvieae*, highlighting their antimicrobial potential. We used an LC–MS/MS-based computational untargeted metabolomics and cutting-edge cheminformatics tools as well as manual dereplication strategies to chemically profile 26 most active extracts, and annotated compound classes such as bile acids, diketopiperazines, indole alkaloids and lipids. Many peak ions remained unannotated, suggesting the presence of new bioactive molecular families. These findings highlight the bioprospecting potential of cultivable bacteria associated with mesopelagic fauna.

**Graphical Abstract:**

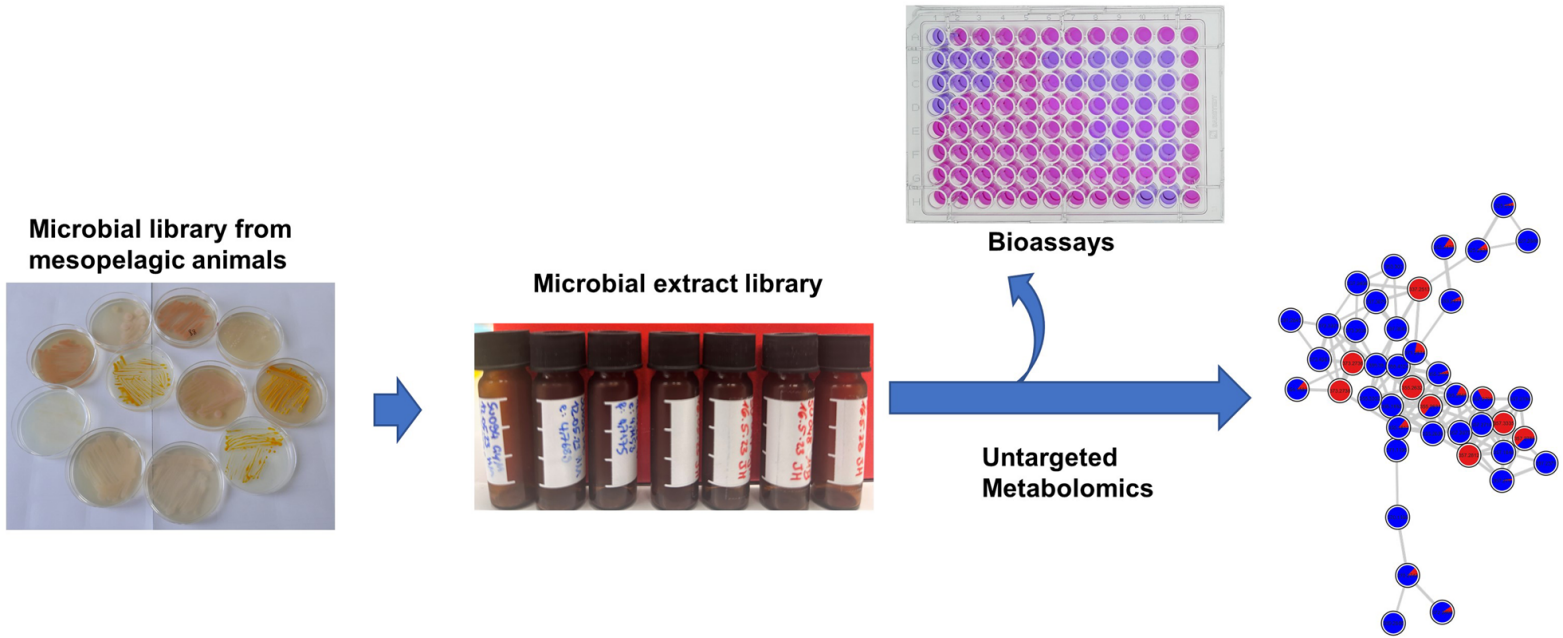

**Supplementary Information:**

The online version contains supplementary material available at 10.1007/s13659-025-00527-6.

## Introduction

Marine microbial symbionts play a crucial role in numerous ecosystems, establishing intricate relationships with their host organisms. These symbiotic interactions, whether mutualistic, commensal, or parasitic, considerably influence health and disease states and ecological balance of their hosts [1]. This dynamic interplay between hosts and their symbionts has emerged as a focal point for scientific exploration, unlocking the vast potential of symbionts in biotechnology and medicine as many marine-derived molecules, already approved as human medicines or in the clinical trials, originate from symbiotic microorganisms [2, 3]. Marine fish harbor complex assemblies of microbial communities, particularly in the gut, gills, and skin. The gut microbiome, due to the high nutrient density of the intestines, is thought to be one of the most concentrated bacterial communities in the otherwise dilute marine environment [4]. The skin and gills, constantly exposed to surrounding seawater, interact dynamically with microorganisms, influencing the overall microbiota of fish. These microbes are essential for the host’s health, affecting key physiological processes such as digestion, immune function, epithelial proliferation and pathogen defense [5]. Additionally, microbial communities associated with fish serve as a valuable source of enzymes, probiotics, as well as secondary metabolites, with antimicrobial, anticancer, and immunomodulatory activities, hence being explored increasingly for their potential in biotechnology and medicine [6, 7]. For example, bacteriocin CAMT6, a small peptide produced by *Enterococcus durans* YQ-6 isolated from the fish *Larimichthys polyactis* in the South China Sea, effectively inhibited the biofilm-forming pathogen *Listeria monocytogenes* by disrupting its cell membrane permeability [8]. Similarly, sebastenoic acid, a bioactive lipid isolated from the bacterium strain FI-1004 sourced from the gut of a fish, demonstrated potent antimicrobial activity against human pathogens *Bacillus subtilis*, *Staphylococcus aureus*, *Enterococcus faecium*, and *Vibrio mimicus* [9]. A series of unprecedented phenylpropanoid piperazines (chrysosporazines) that reverse the doxorubicin resistance in P-glycoprotein overexpressing human colon carcinoma cells have been reported from the fungus *Chrysosporium* sp. sourced from the gastrointestinal track of the Australian mullet fish [10, 11] Despite their biotechnological potential, fish microbiomes remain largely underexplored, particularly in the mesopelagic zone, as most studies have focused on commercially important fish or those from shallow waters [9, 12].

The mesopelagic zone (or twilight zone) of the ocean, spanning depths between 200 to 1000 m, is a unique and challenging ecosystem. This expansive habitat harbors a rich tapestry of animal life, including invertebrates such as squids, cuttlefish, and various jellyfish species alongside distinct fish species of several families, such as Myctophidae (lanternfish) and Sternoptychidae (hatchetfish or pearlside) [13–15]. These mesopelagic fish are the most abundant vertebrates in marine environments [16]. They exhibit several morphological features in order to adapt to challenging abiotic conditions in their habitat, such as reduced body size, complex eyes for improved vision in dim light, bioluminescence organs for counterillumination, aiding in predator evasion and prey attraction and microbial association [17, 18]. Extreme conditions in the mesopelagic zone, such as low temperatures, high hydrostatic pressure, and specific photoperiods, exert also selective pressure on microbial communities, driving the evolution of unique biochemical adaptations, including the biosynthesis of structurally distinct and bioactive molecules. This positions these microbes as promising candidates for bioprospecting and the discovery of novel natural products [19]. Due to challenges of access and the resource-intensive nature of sampling, microorganisms associated with animals living in one of the largest and harshest deep-sea environment are underexplored. Only a few studies have investigated the microbiome of deep-sea fish and their antimicrobial potential [4, 20–22]. A metagenomics study of the gut of deep-sea fish caught off the coast of Iceland identified dominant phyla, including Proteobacteria, Actinomycetota and Bacteriodota [4], revealing new microbial species and a notable scarcity of antibiotic resistance genes [4]. Another study screened antimicrobial activity of bacterial isolates recovered from the gut of deep-sea fish using the colony overlay assays, and demonstrating antimicrobial activity against a range of pathogens [21]. However, information on metabolome or broader bioactivity potential of these microbes remains scarce.

In this study, we explored the application potential of microorganisms associated with mesopelagic fish, as well as few other animals (such as jellyfish, squid, krill) and surrounding seawater from the North Atlantic Ocean, for pharmaceutical and aquacultural industries. Using a culture-dependent approach, we isolated and identified 643 bacterial colonies from these diverse sources. Phylogenetically distinct isolates were selected and cultivated in two growth media. Ethyl acetate extracts were screened for in vitro bioactivities, including antimicrobial (against 10 human and fish pathogens), anticancer (2 cancer cell lines), and for general toxicity. The most active broad-spectrum antimicrobial extracts were selected and analyzed by LC–MS/MS-based untargeted metabolomics, employing the feature-based molecular networking (FBMN) and other tools from SIRIUS suite [23, 24]. This approach enabled prioritization of microbes for future downstream chemical analysis, including purification and characterization of novel antimicrobial natural products. Our study highlights the potential of mesopelagic fauna-associated microorganisms as sources of bioactive compounds and underscores the potential of these communities for applications in the pharmaceutical and agricultural sectors.

## Results

### Mesopelagic animals and their cultivable bacteria

Fish specimens, representing 13 different species, were collected from the mesopelagic zone of the North Atlantic Ocean across 12 sampling stations (Table 1, Fig. S1). The lanternfish *Myctophum punctatum* was sampled at two stations (427 and 433). Two other lanternfish species *Notoscopelus kroyeri* and *Benthosema glaciale* were collected from multiple stations as shown in Table 1. All other fish species were exclusively sourced from one single station (Table 1). Other mesopelagic animals included the krill species *Meganyctiphanes norvegica*, the squid *Gonatus* sp., and the jellyfish *Atolla* sp., all originating from the same station (439).

**Table 1:** Samples obtained in the North Atlantic; their geographical location (in: start of trawling, out: stop of trawling), the organism and tissue used for microbial isolation, and the sampling depth

Station No	Coordinates	Sample type	Tissue	Depth
414	in: 63°12.93 N; 18°53.79 W out: 63° 09.46 N; 18°53.57 W	Fish: *Notoscopelus kroyeri,* *Benthosema glaciale* *Stomias boa ferox* *Chauliodus sloani* Water: 325 m	Gill & gut Gill & gut Gut Gut Seawater	300–350 m
424	in: 60°48.04 N; 28°57.15 W out: 60°47.77 N; 29°57.15 W	Fish: *Notoscopelus kroyeri*	Gill & gut	400–440 m
427	in: 60°36.58 N; 32°29.38 W out: 60°35.48 N; 32°33.27 W	Fish: *Notoscopelus kroyeri* *Benthosema glaciale* *Myctophum punctatum* *Arctozenus rissoi* Water: 325 m	Gill & gut Gill & gut Gill & gut Gill Seawater	200–300 m
433	in: 58° 53.80 N; 40°49.58 W out: 58°54.06 N; 40°41.71 W	Fish: *Notoscopelus kroyeri* *Benthosema glaciale* *Myctophum punctatum* Water: 425 m	Gill & gut Gill & gut Gill & gut Seawater	400–450 m
439	in: 61°36.75 N; 35°32.69 W out: 61°36.49 W, 35°38.49 W	Fish: *Cyclothone microdon* Squid: *Gonatus* sp. Jellyfish: *Atolla* sp. Krill: *Meganyctiphanes norvegica*	Gill & gut Surface, intestine & ink sac Inner & outer umbrella Surface	1000–0 m
448	in: 61°53.24 N; 23°28.55 W out: 61°53.23 W, 23°32.55 W	Fish: *Protomyctophum arcticum*	Gill & gut	280–310 m
449	in: 61°53.57 N; 23°34.12 W out: 61°53.90 N; 23°40.84 W	Fish: *Scopelogadus beanii*	Gill & gut	570–625 m
451	in: 61°53.96 N; 23°46.22 W out: 61°53.23 N; 23°53.96 W	Fish: *Bathylagus euryops*	Gill, gut & skin	1000–0 m
452	615,408 N; 21,051 W	Fish: *Paralepis coregonoides*	Gill & gut	Surface, evening catch
458	in: 61 46.46 N; 16 53.44 W out:61 46. 41 N; 16 57.11 W	Fish: *Maurolicus muelleri*	Gill & gut	260 m
462	in: 62°57.05 N; 21°29.73 W out: 62°53.21 W, 21°32.36 W	Fish: *Xenodermichthys copei*	Gill, gut & skin	1000 m

**Fig. 1:**
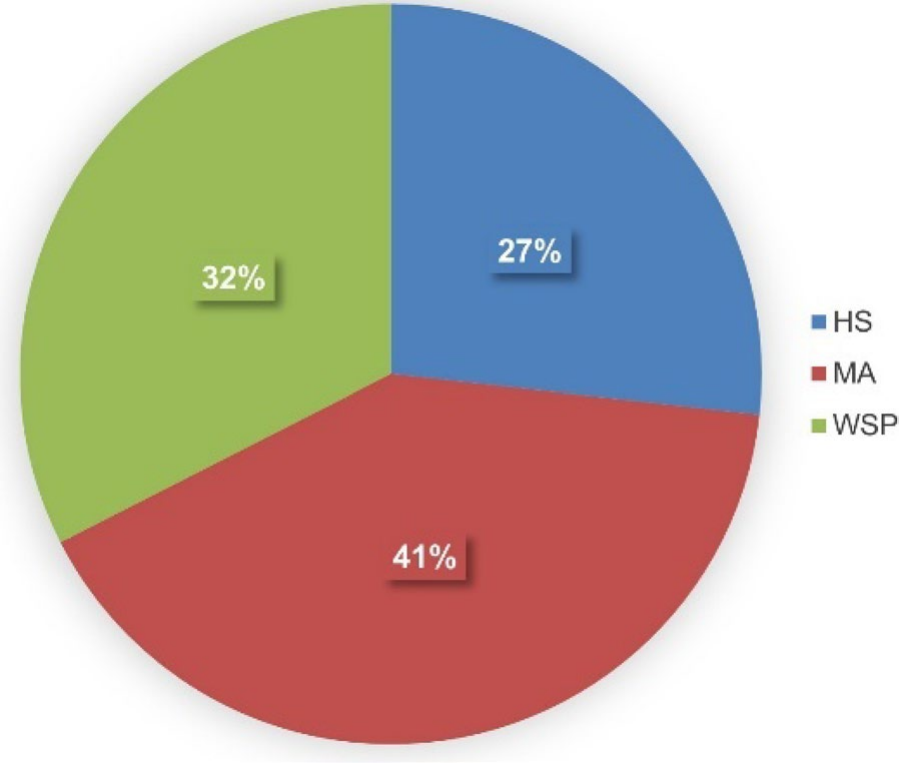
Distribution of 643 bacterial isolates from mesopelagic animals and seawater obtained from the North Atlantic Ocean. Isolation media: Hastings (HS), Marine Agar (MA), Wickerham (WSP)

Using three isolation media, namely Hastings (HS), Marine agar (MA), and Wickerham (WSP), we retrieved 643 bacterial isolates originating from different animal sources and seawater (Table 1, Table S1). Notably, no fungi were isolated, although WSP medium is optimized for eutrophic conditions and typically support fungal growth. The HS medium yielded 27% of the bacterial isolates, MA medium contributed 41%, and WSP medium accounted for 32% of the total isolates (Fig. 1). All isolates were unambiguously identified through a comprehensive BLAST comparison of their 16S rRNA gene sequences against the NCBI nucleotide database (Table S2).

**Fig. 2:**
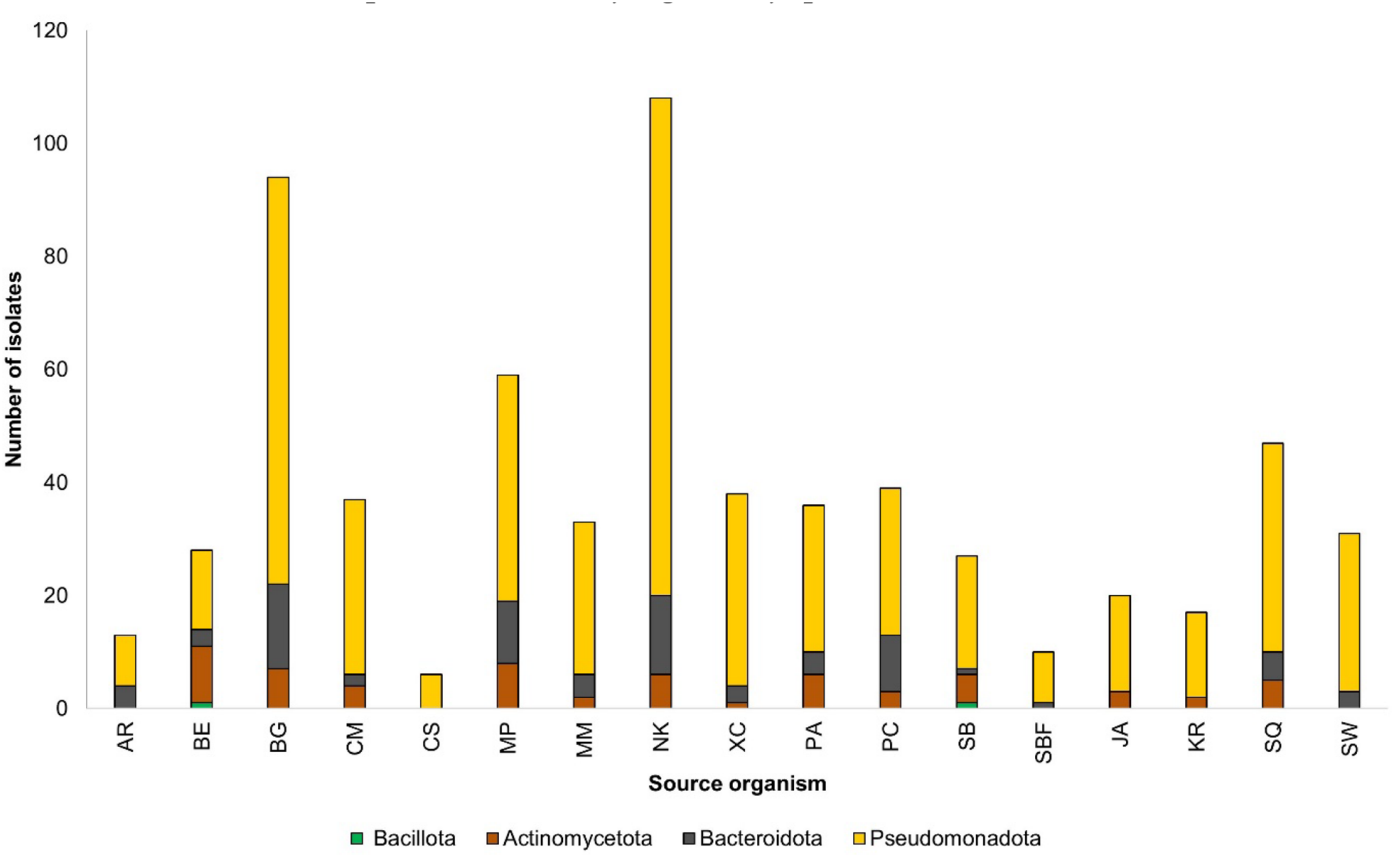
Diversity of isolated bacteria at phylum level from mesopelagic fish species, *Arctozenus rissoi* (AR), *Bathylagus euryops* (BE), *Benthosema glaciale* (BG), *Cyclothone microdon* (CM), *Chauliodus sloani* (CS), *Myctophum punctatum* (MP), *Maurolicus muelleri* (MM), *Notoscopelus kroyeri* (NK), *Protomyctophum arcticum* (PA), *Paralepis coregonoides* (PC), *Scopelogadus beanii* (SB), *Stomias boa ferox* (SBF), *Xenodermichthys copei* (XC), jellyfish *Atolla* sp. (JA), krill *Meganyctiphanes norvegica* (KR), squid *Gonatus* sp. (SQ), and seawater (SW)

The 643 identified isolates represented the phyla Actinomycetota, Bacillota, Bacteroidota, and Pseudomonadota (Fig. 2). The Gram-negative phylum Pseudomonadota dominated the isolates from all sources. Isolated bacteria mainly belong to three classes, Gammaproteobacteria, Alphaproteobacteria and Epsilonproteobacteria (Table S2). Except for the dragonfish *Chauliodus sloani*, all sampled mesopelagic organisms and seawater provided cultivable bacteria belonging to at least two phyla. Lanternfish species *Notoscopelus kroyeri* and *Benthosema glaciale* exhibited highest abundance of microbial communities. Notably, representatives of the Gram-positive phyla Actinomycetota and Bacillota were completely absent from the seawater samples. The only Bacillota representative identified in this study was the Gram-positive *Planococcus* sp. isolated from the gut of the dragonfish *Scopelogadus beanii* and the skin of the deep-sea smelt *Bathylagus euryops*.

**Fig. 3:**
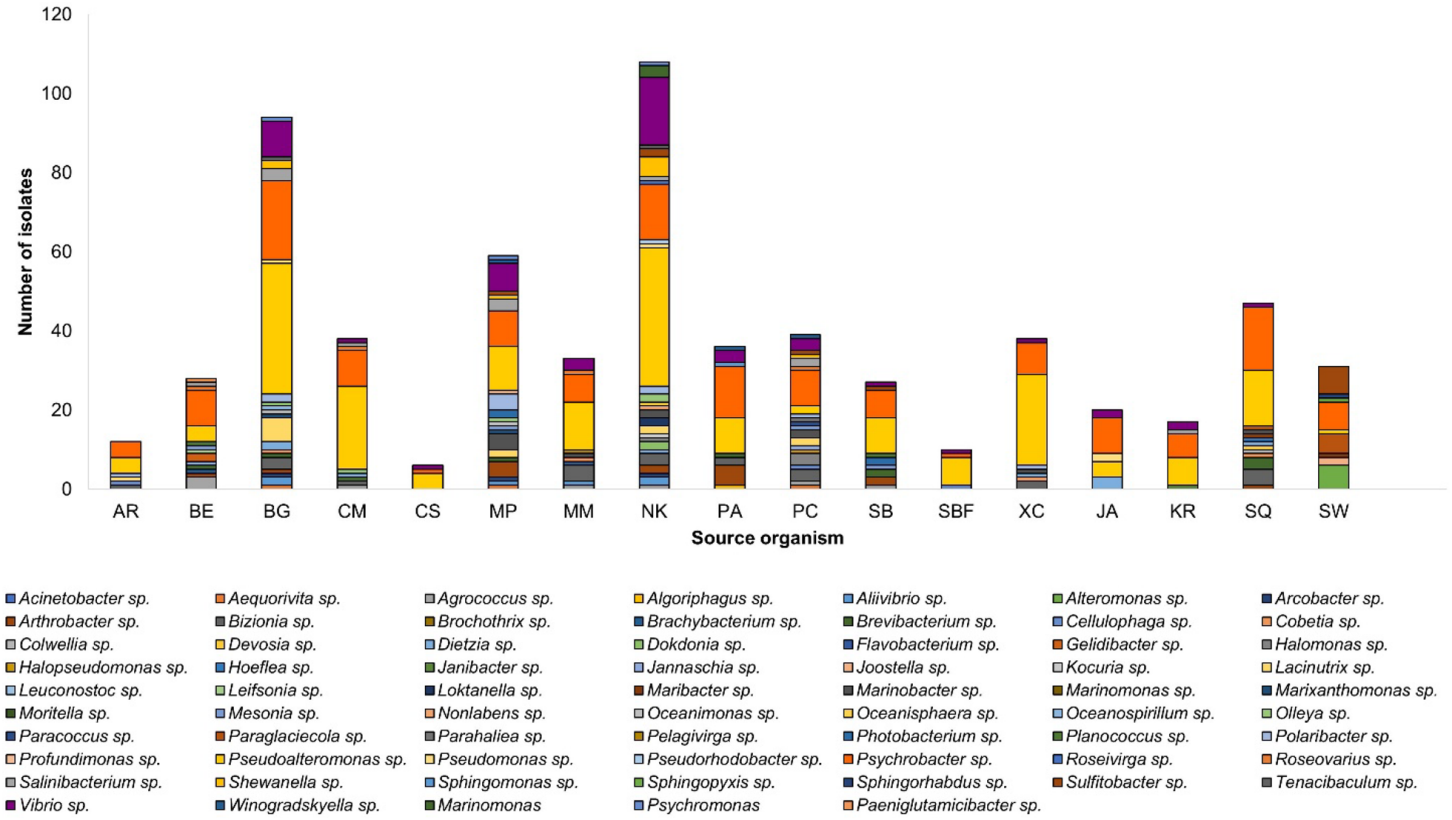
Diversity of cultivable bacteria at genus level in fish; *Arctozenus rissoi* (AR), *Bathylagus euryops* (BE), *Benthosema glaciale* (BG), *Cyclothone microdon* (CM), *Chauliodus sloani* (CS), *Myctophum punctatum* (MP), *Maurolicus muelleri* (MM), *Notoscopelus kroyeri* (NK), *Protomyctophum arcticum* (PA), *Paralepis coregonoides* (PC), *Scopelogadus beanii* (SB), *Stomias boa ferox* (SBF), *Xenodermichthys copei* (XC), jellyfish *Atolla* sp. (JA), krill *Meganyctiphanes norvegica* (KR), squid *Gonatus* sp. (SQ), and Seawater (SW)

Cultivable bacteria exhibited a high degree of diversity at genus level, with 55 genera being identified across the mesopelagic animals and seawater samples (Fig. 3, Table S2). The genus *Pseudoalteromonas* emerged as consistently abundant and widespread in the mesopelagic zone. The broad distribution of *Psychrobacter* and *Vibrio* spp. indicates their adaptability to diverse ecological conditions. Other genera deriving from at least five mesopelagic animals include Gram-positive *Agrococcus* sp., *Arthrobacter* sp.*, Brevibacterium* sp., *Dietzia* sp. and *Salinibacterium* sp., and Gram-negative *Bizonia* sp., *Marinobacter* sp., *Polaribacter* sp., and *Sulfibacter* sp.

In contrast, certain bacterial species were retrieved only from particular animal species, emphasizing potential niche preferences (Table S2). For example, Gram-negative bacteria *Paracoccus* sp., *Parahaliea* sp., *Halopseudomonas* sp. and *Jannaschia* sp. were exclusively isolated from the barracudina fish *Paralepis coregonoides* while *Colwellia* sp, *Devosia* sp. and *Hoeflea* sp. were exclusive to the squid *Gonatus* sp. Other bacterial isolates sourced from a single host include Gram-negative *Pelagivirga* sp. (pearlside *Maurolicus muelleri*); Gram-negative *Gelidibacter* sp. and Gram-positive *Leifsonia* sp. (deep-sea smelt *Bathylagus euryops*)*;* Gram-positive *Janibacter* sp.*,* Gram-negative *Algoriphagus* sp., and *Sphingomonas* sp. (lanternfish *Protomyctophum arcticum*).

Considering the sampling sites (Table 1), stations 427 and 439 yielded the highest number of isolates (Fig. S2). The bacterial diversity associated with the widely distributed lanternfish *M. punctatum*, *N. kroyeri*, and *B. glaciale*, revealed a complex microbial landscape. *Notoscopelus kroyeri* had the highest bacterial abundance at station 424, *B. glaciale* at station 414, and *M. punctatum* at station 427. Although collected from different stations, common genera such as Gram-positive *Salinibacterium,* Gram-negative *Aliivibrio* sp.*, Sulfitobacter* sp.*, Arcobacter* sp.*, Olleya* sp. and *Shewanella* sp. were present in all three lanternfish specimens. Gram-negative *Profundimonas* sp. and *Oceanospirillum* sp. were unique to *M. punctatum* and *B. glaciale*, respectively. The third lanternfish *N. kroyeri* displayed the most unique microbial genera among all sampled animals. These include Gram-negative *Dokdonia* sp., *Loktanella* sp., *Nonlabens* sp., *Oceanisphaera* sp., *Pseudorhodobacter* sp., *Roseivirga* sp., *Marinomonas* sp. and Gram-positive *Kocuria* sp. Analysis of the cultivable microbial communities across fish tissues revealed the gills to host higher diversity than the gut and the skin (Fig. S3). It is important to note that isolates from fish skin were limited to only few fish individuals in this study due to severe skin damage during trawling.

## Antimicrobial and anticancer activity of the microbial extracts

Out of the 643 identified isolates, 394 were selected based on their biological safety level (exclusion of BSL-2 organisms), host tissue of origin, sampling location, and phylogeny. Selected isolates were grown in two liquid media; the nutrient-poor marine broth (MB) and the nutrient-rich glucose–yeast–malt (GYM), and extracted with ethyl acetate (EtOAc) after 7 days of cultivation. The discrepancy between the number of isolates submitted for cultivation (788) and the number of extracts obtained (590) is because 198 isolates did not grow in the selected culture media. Labelling of extracts is based on the taxonomical ID, strain number (Table S2) and the culture medium (MB or GYM). Hence, 590 bacterial extracts were assessed for their in vitro antimicrobial activity against eight human pathogens (Table S3); i) the ESKAPE panel that comprises six highly virulent and drug-resistant bacterial pathogens (*Enterococcus faecium*, methicillin-resistant *Staphylococcus aureus* (MRSA), *Klebsiella pneumoniae*, *Acinetobacter baumannii*, *Pseudomonas aeruginosa*, *Escherichia coli*), ii) two human pathogenic yeasts (*Candida albicans*, *Cryptococcus neoformans*). Two cancer cell lines (human melanoma cell line A-375 and colon cancer cell line HCT-116) were utilized to assess the antitumor activity of the extracts. A general toxicity test using a non-cancerous human keratinocyte cell line HaCaT was also added to the screening panel. Given that the extracts originated mainly from fish symbionts, we hypothesized that they might contain compounds that inhibit the growth of fish pathogens. This potential benefit for aquaculture applications prompted further screening of these extracts against two fish pathogens *Lactococcus garvieae* and *Vibrio ichthyoenteri*.

**Fig. 4:**
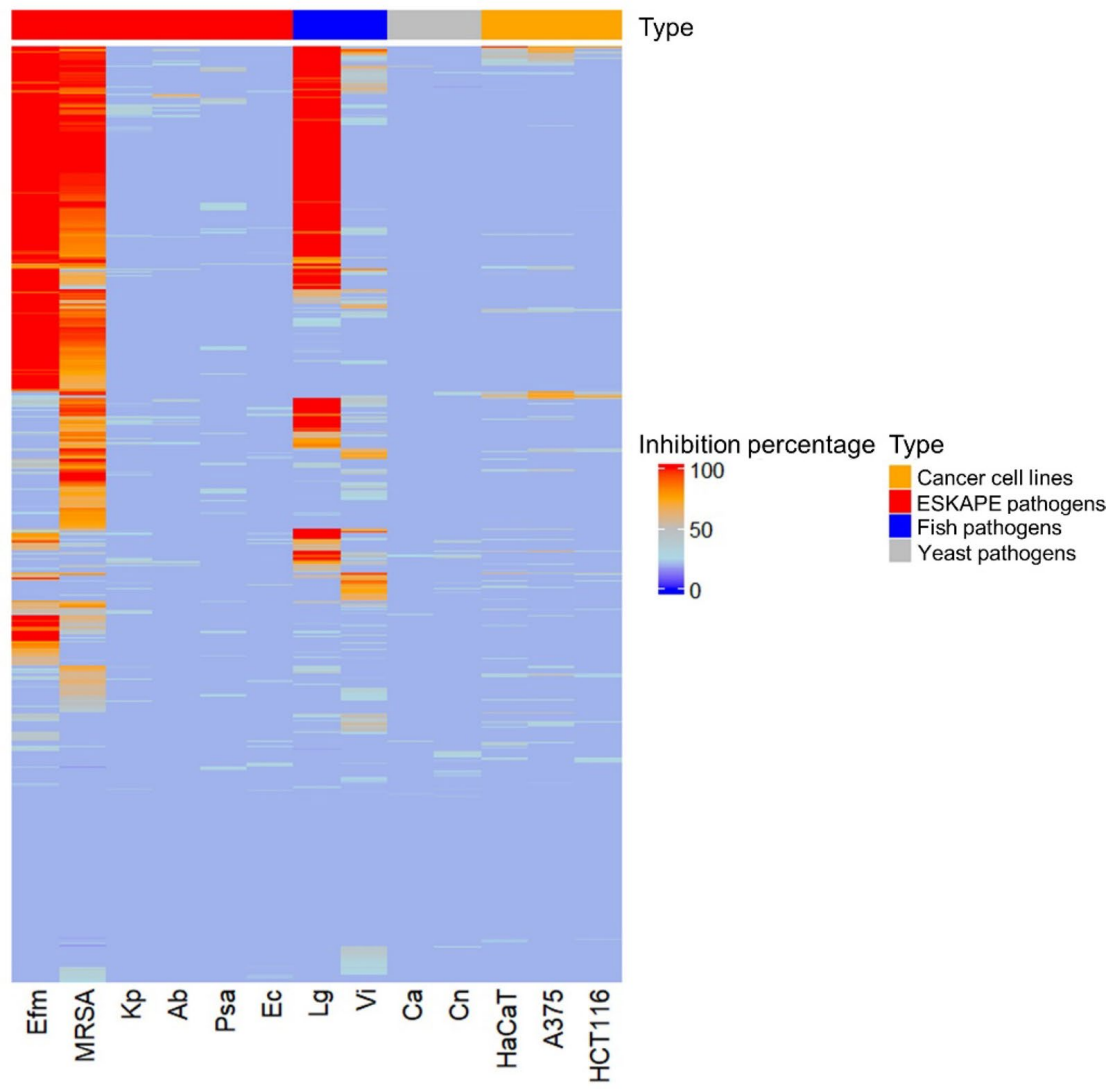
Heatmap showing antimicrobial activity (% inhibition) of bacterial extracts at a test concentration of 100 μg/mL against test pathogens and cancer cells. Ca: *Candida albicans*, Cn: *Cryptococcus neoformans*, Efm: *Enterococcus faecium*, MRSA: methicillin-resistant *Staphylococcus aureus*, Kp: *Klebsiella pneumoniae*, Ab: *Acinetobacter baumannii*, Psa: *Pseudomonas aeruginosa*, Ec: *Escherichia coli*, Lg: *Lactococcus garvieae*, Vi: *Vibrio ichthyoenteri*, A-375: human melanoma cell line, HCT-116: colon cancer cell line, HaCaT: human keratinocyte cell line

The extract library was screened in multiple bioassays at a concentration of 100 µg/mL (Fig. 4, Table S4). Notably, approximately 60% of the extracts showed considerable growth inhibition (70% or more) against at least one pathogen at this concentration. As displayed in Fig. 4, two Gram-positive human bacterial pathogens *E. faecium*, MRSA and the fish pathogenic bacterium *L. garvieae* demonstrated the highest susceptibility to the extracts (activity > 70%). While a larger number of extracts (274) inhibited MRSA, with most showing activities ranging from 70 to 90%, more potent antimicrobial activity was observed against *E. faecium* (246 extracts) and *L. garvieae* (194 extracts), where the majority demonstrated 100% inhibition (Table S4). All other pathogens, as well as the cancer cell lines, displayed minimal susceptibility to all extracts (Table S4). The human cell line HaCaT was largely unaffected suggesting the non-toxic nature of the extracts.

Due to very large sample numbers, only the extracts significantly inhibiting the growth (> 80%) of the three most susceptible pathogens (*E. faecium*, MRSA, and *L. garvieae*) were subjected to IC_50_ determination. Table 2 displays the 26 most potent extracts with IC_50_ values < 20 µg/mL against at least one of the susceptible test pathogens. These extracts were primarily derived from Gammaproteobacteria, followed by Actinomycetes and then Flavobacteriia. Approximately 56% of the most potent extracts were derived from the nutrient-rich GYM medium (Table 2). Mesopelagic fish associated bacteria demonstrated stronger and broader antibacterial activities compared to other isolation sources, such as squid and krill. Except for three isolates sourced from the surface and intestines of the squid *Gonatus* sp., all active extracts originated from the gills and gut of the following fish species: *N. kroyeri* (10 isolates), *S. boa ferox* (3 isolates), *B. euryops* (2 isolates), *M. punctatum* (2 isolates)*, B. glaciale* (2 isolates), *M. muelleri* (2 isolates) and one isolate each from *X. copei, S. beanii* and *C. sloani* (Table 2).

**Table 2:** Antimicrobial activity of 26 extracts with IC_50_ value ≤ 20 µg/mL against at least one test pathogen

Extract	Bacterial Class	Origin	Source organism	Activity (IC_50_ values)			Tissue
				Efm	MRSA	Lg	
*Shewanella* sp. SU082MB	Gammaproteobacteria	Fish	*Notoscopelus kroyeri*	20.0	24.2	19.7	Gut
*Pseudoalteromonas* sp. SU085aMB	Gammaproteobacteria	Fish	Stomias boa ferox	51.5	24.3	19.5	Gut
*Psychrobacter* sp. SU087GYM	Gammaproteobacteria	Fish	Stomias boa ferox	36.1	17.8	17.9	Gut
*Pseudoalteromonas* sp. SU088GYM	Gammaproteobacteria	Fish	Stomias boa ferox	19.0	14.5	10.9	Gut
*Pseudoalteromonas* sp. SU096MB	Gammaproteobacteria	Fish	*Chauliodus sloani*	15.6	12.3	19.8	Gut
*Pseudoalteromonas* sp. SU286GYM	Gammaproteobacteria	Fish	*Notoscopelus kroyeri*	19.9	7.9	17.7	Gills
*Pseudoalteromonas* sp. SU286MB	Gammaproteobacteria	Fish	*Notoscopelus kroyeri*	29.0	30.0	19.9	Gills
*Pseudoalteromonas* sp. SU289MB	Gammaproteobacteria	Fish	*Notoscopelus kroyeri*	25.0	19.2	19.6	Gills
*Halomonas* sp. SU293GYM	Gammaproteobacteria	Fish	*Notoscopelus kroyeri*	72.0	13.7	100	Gills
*Psychromonas* sp. SU383MB	Gammaproteobacteria	Fish	*Benthosema glaciale*	17.7	32.4	28.3	Gills
*Pseudoalteromonas* sp. SU391GYM	Gammaproteobacteria	Fish	*Benthosema glaciale*	19.1	18.1	14.3	Gills
*Dokdonia* sp. SU422MB	Flavobacteriia	Fish	*Notoscopelus kroyeri*	17.7	15.5	12.5	Gills
*Shewanella* sp. SU423GYM	Gammaproteobacteria	Fish	*Notoscopelus kroyeri*	20.4	22.4	19.5	Gills
*Agrococcus* sp. SU436GYM	Actinomycetes	Fish	*Notoscopelus kroyeri*	9.7	12.4	7.1	Gut
*Bizionia* sp. SU438MB	Flavobacteriia	Fish	*Notoscopelus kroyeri*	57.3	62.1	18.3	Gut
*Paeniglutamicibacter* sp. SU794GYM	Actinomycetes	Fish	*Scopelogadus beanii*	11.6	13.8	11.5	Gills
*Pseudoalteromonas* sp. SU449GYM	Gammaproteobacteria	Fish	*Myctophum punctatum*	23.0	14.0	24.0	Gills
*Pseudoalteromonas* sp. SU456GYM	Gammaproteobacteria	Fish	*Myctophum punctatum*	22.0	13.0	24.0	Gills
*Marinobacter* sp. SU597MB	Gammaproteobacteria	Squid	*Gonatus* sp.	31.0	15.0	36.0	Surface
*Psychrobacter* sp. SU607MB	Gammaproteobacteria	Squid	*Gonatus* sp.	> 100	19.0	> 100	Intestine
*Brevibacterium* sp. SU651MB	Actinomycetes	Squid	*Gonatus* sp.	34.0	7.0	11.0	Intestine
*Arthrobacter* sp. SU809GYM	Actinomycetes	Fish	*Bathylagus euryops*	10.0	23.0	46.0	Gills
*Paeniglutamicibacter* sp. SU836GYM	Actinomycetes	Fish	*Bathylagus euryops*	7.0	11.0	12.0	Skin
*Psychrobacter* sp. SU841GYM	Gammaproteobacteria	Fish	*Maurolicus muelleri*	53.0	18.0	> 100	Gills
*Pseudoalteromonas* sp. SU930MB	Gammaproteobacteria	Fish	*Xenodermichthys copei*	65.0	11.0	> 100	Skin
*Agrococcus* sp. SU880GYM	Actinomycetes	Fish	*Maurolicus muelleri*	23.0	8.0	32.0	Gut
Positive control	–	–	–	1.1	4.2	2.0	–

*Efm* *Enterococcus faecium*, *MRSA* methicillin-resistant *Staphylococcus aureus*, *Lg* *Lactococcus garvieae*. Positive controls: ampicillin (Efm and Lg), chloramphenicol (MRSA). Extract name combines the taxonomic ID, the strain number and the culture medium, MB or GYM

Extracts of *Psychromonas* sp. strain SU383MB (from gills of *B. glaciale*) and *Arthrobacter* sp. strain SU809GYM (from gills of *B. euryops*), exhibited substantial activity against *E. faecium*, with IC_50_ values of 17.7 µg/mL and 10 µg/mL, respectively. Extracts of multiple *Pseudoalteromonas* strains, including SU088GYM (from gut of *S. boa ferox*), SU286GYM (from gill of *N. kroyeri*), SU096MB (from gut of *C. sloani*) and SU391GYM (from gill of *B. glaciale*), along with *Dokdonia* sp. isolate SU422MB (from gill of *N. kroyeri*), *Agrococcus* sp. SU436GYM (from gut of *N. kroyeri*), *Paeniglutamicibacter* sp. SU794GYM (from gill of *S. beanii*) and *Paeniglutamicibacter* sp. SU836GYM (from skin of *B. euryops*) displayed the most potent and the broadest spectrum of activity against all three pathogens with IC_50_ values ranging from 7.0–19.8 µg/mL. Some extracts also selectively exhibited potent activities against MRSA and the fish pathogen *L. garvieae*. These include extracts of *Pseudoalteromonas* sp. SU289MB, and *Halomonas* sp. SU293GYM (from gills of lanternfish *N. kroyeri*), *Agrococcus* sp. SU880GYM (from gut of pearlside *M. muelleri*), and *Brevibacterium* sp. SU651MB (from intestine of the squid *Gonatus* sp., Table 2). Notably, the gills of lanternfish *N. kroyeri* emerged as the tissue source harboring the highest number of bioactive isolates.

## Metabolomics

**Fig. 5:**
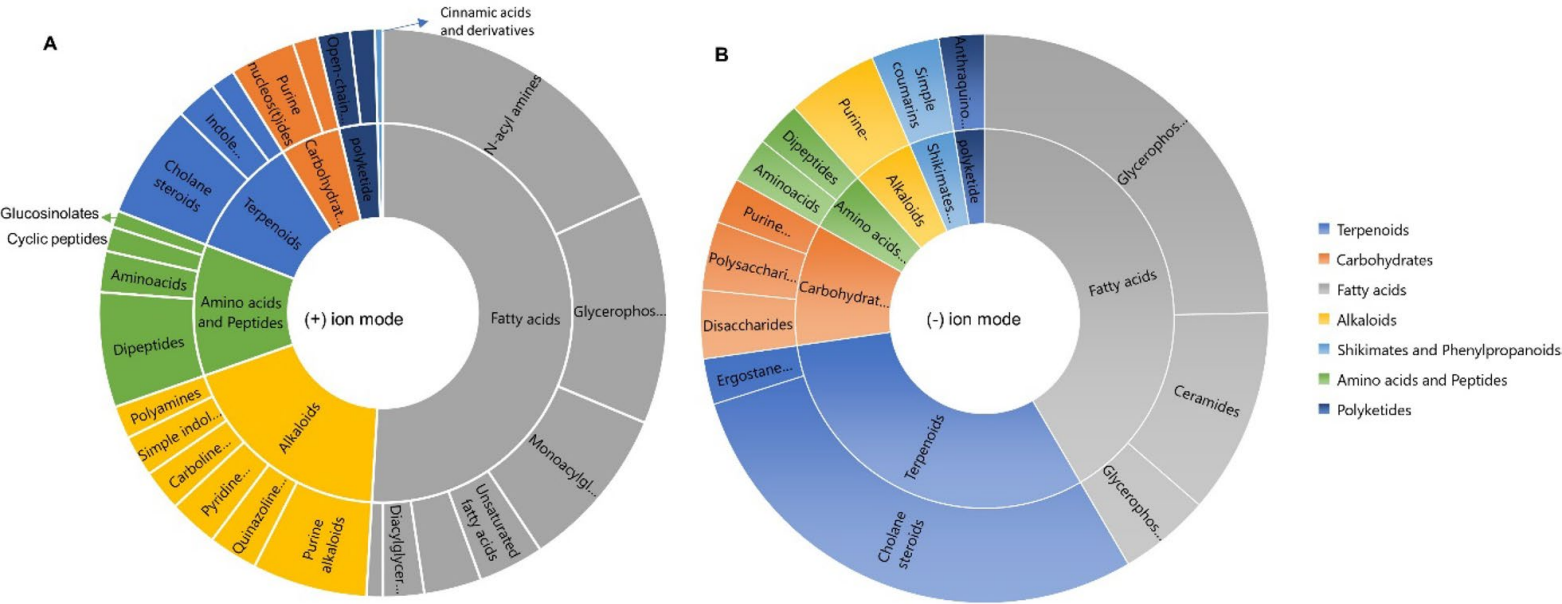
Chemical classes and pathways (based on NPClassifier ontology) of the unique library compounds annotated with cosine score > 0.9 from (A) ( +) ionization polarity mode and (B) (−) ionization polarity mode, based on CANOPUS compound class prediction

In order to comparatively analyse the metabolomes of the bacteria associated with the mesopelagic fauna, the most promising 26 extracts were submitted for computational untargeted metabolomics study using feature-based molecular networking (FBMN) [23] and several tools from the SIRIUS suite, e.g., CANOPUS and CSI:FingerID tools [24]. These tools facilitate metabolite annotations significantly. The spectral data set obtained from MZmine was submitted to SIRIUS to obtain accurate molecular formulae, and structural annotations through CSI: FingerID [25], followed by the CANOPUS chemical class assignment. CANOPUS predicts compound classes directly from MS/MS data without relying on any database, enabling classification of unknown compounds [26]. It uses Natural Product Classifier (NPClassifier) which organizes the compounds into hierarchical levels, including Pathway, Superclass, and Class [27]. Based on CANOPUS, the 26 most bioactive extracts revealed a diverse array of predicted compound classes across multiple pathways (Table S5). A summary of the compound classes predicted (score > 0.9 at all classification levels) are displayed at ‘Pathway’ and ‘Class’ level in Fig. 5.

In the positive ion mode (Fig. 5A), fatty acids (or lipids) emerged as predominant with a notable number of classes identified, with N-acyl amines, glycerophosphoethanolamines and monoacylglycerols being the most dominant. Other lipid types included some unsaturated fatty acids, diacylglycerols and N-acyl glycerols. Terpenoids also featured prominently, and cholane steroids and indole-diterpenoids were the main classes annotated. Alkaloids were abundant as well, and we could predict a number of purine-related compounds, pyridine and quinazoline derivatives. Other alkaloids included polyamines, indole and carboline alkaloids. The amino acids and peptides predicted included dipeptides, amino acids, and cyclic peptide type compounds. Open-chain polyketides and some catechol-like compounds represented Polyketides. The shikimate and phenylpropanoid type phenolics were minimally represented with only cinnamic acids and their derivatives. Purine and pyrimidine nucleosides were the only compound class predicted to contain carbohydrates.

In the negative ion mode (Fig. 5B), a slightly different pattern was observed. Although both polarities revealed fatty acids for the mostly Gram-negative bacterial extracts, the next most abundant pathway was the terpenoids represented by cholane steroids and ergostane steroids. Carbohydrates featured polysaccharides, disaccharides, and purine nucleosides. Several compound classes observed in the (+) ion mode as alkaloids, shikimates and phenylpropanoids, amino acids and peptides, were also predicted in the negative ion mode (Fig. 5B). Overall, the observation of diverse range of chemical classes underscores the broad biosynthetic capabilities of mesopelagic bacteria.

**Fig. 6:**
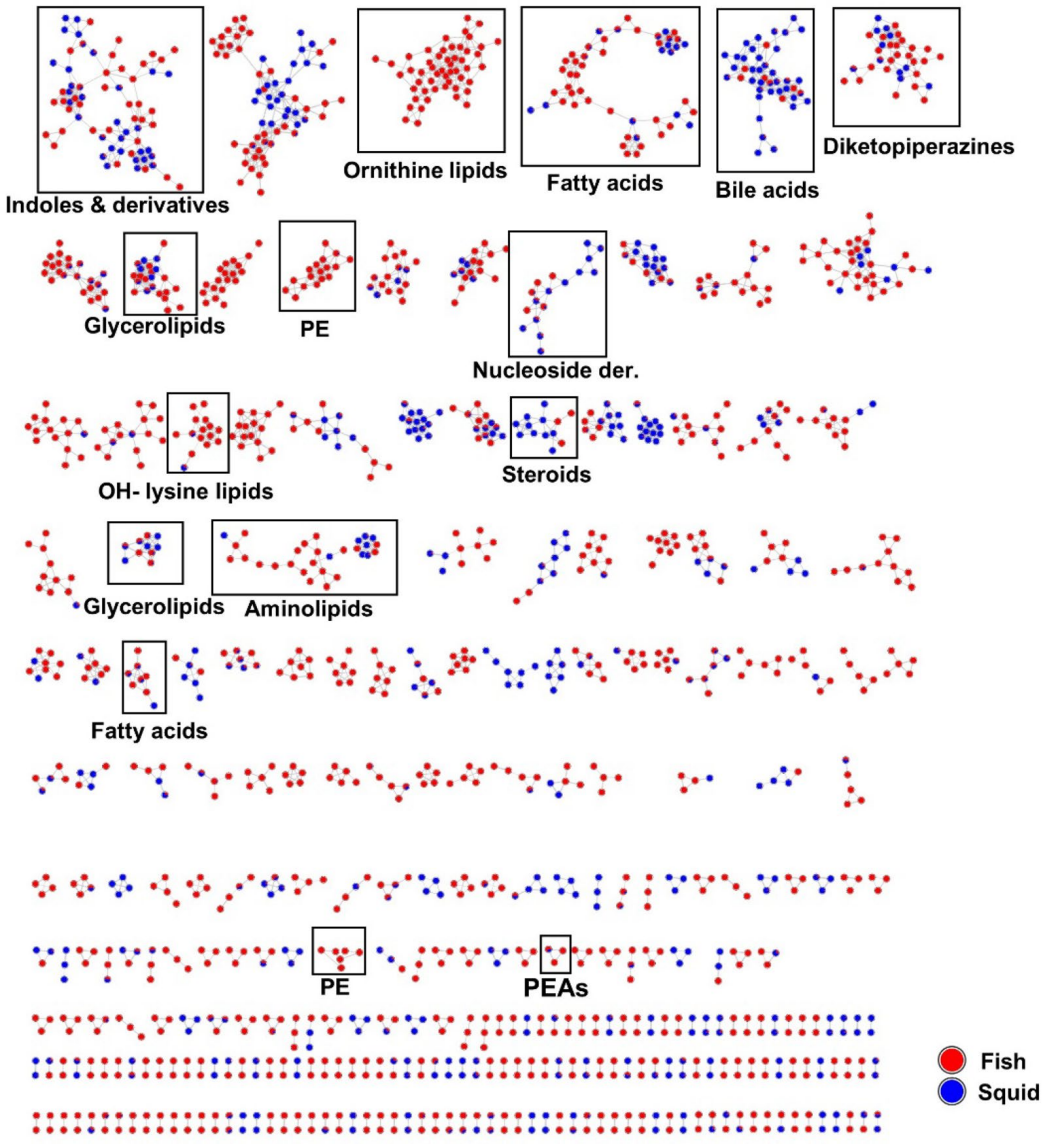
Molecular network generated from UPLC-( +)-ESI–MS/MS data of 26 active bacterial extracts according to isolation source (fish or squid). PE: phosphatidylethanolamines, PEAs: phenylethylamides

An in-depth metabolome analysis was conducted using feature-based molecular networking (FBMN), which clusters ions with similar MS/MS spectra to enhance dereplication [23]. The extracts were analyzed not only for their chemical space, but also to unravel molecular families potentially contributing to their potency. Manual dereplication against databases such Dictionary of Natural Products, MarinLit, SciFinder, Natural Product Atlas and COCONUT was also conducted. All annotations were displayed in the molecular network. In total, 1669 nodes (features) were organized into 139 clusters, each containing more than two nodes per cluster (Fig. 6). Many of these clusters were common to multiple extracts. Few unannotated clusters were unique to some extracts such as *Paeniglutamicibacter* sp. SU794GYM, *Pseudoalteromonas* sp. SU088GYM and *Psychrobacter* sp. SU841GYM. Aminolipids, ornithine lipids, fatty acids, and phosphoethanolamines were readily annotated based on their product ion matches to those in the GNPS (Global Natural Products Social Molecular Networking) reference spectral library [28]. These are primary metabolites, which play essential roles as foundational components utilized by microorganisms in diverse metabolic pathways. They were expressed in both cultivation media, MB and GYM (Fig. S4). Although the majority of the 26 bioactive extracts were derived from GYM medium (15 out of 26 extracts), MB medium extracts accounted for 71% of the total features. A total of 35% of the features were common to both culture media (Fig. S4).

Diketopiperazines (small peptides), were also annotated (Fig. 6). Compounds in this cluster include cyclo Leu-Leu (*m/z* 227.1549 [M + H]^+^), cyclo Val-Phe (*m/z* 247.1163 [M + H]^+^), cyclo Phe-4-Hyp (*m/z* 261.1255 [M + H]^+^) and cyclo Pro-Leu (*m/z* 211.1449 [M + H]^+^), cyclo Trp-Pro (*m/z* 284.1650 [M + H]^+^), alongside other dipeptides such as Val-Leu (*m/z* 213.1242 [M + H]^+^), Ile-Tyr (*m/z* 277.1187 [M − H_2_O + H]^+^), phenylalanylglycine (*m/z* 245.0877 [M + Na]^+^). Diketopiperazines have been previously isolated from marine bacteria and fungi [29]. Other nodes with similar masses representing putative isotopes were also observed in this cluster.

The FBMN analysis in the positive ion mode revealed a prominent indole and derivative cluster. Within this cluster some alkaloids were annotated, including tryptophol (*m/z* 162.0918 [M + H]^+^), and adenine (*m/z* 136.0622 [M + H]^+^) type very small molecules. A node annotated as 5-hydroxyindole (*m/z* 134.0605 [M + H]^+^) was solely derived from *Psychrobacter* spp. associated with the gut of fish *S. boa ferox* and intestine of squid *Gonatus* sp. Notably, we putatively identified the β-carboline derivative harmane (*m/z* 183.0774 [M + H]^+^), which is the methylated derivative of norharmane previously reported from a Gram-negative *Lacinutrix* sp. [30] Additional nitrogenous molecules annotated in this cluster included 1-(4-methylphenyl)-9*H*-β-carboline (*m/z* 259.1235 [M + H]^+^), N-acetyltryptamine (*m/z* 203.1184 [M + H]^+^) and N,N-dimethyl-7*H*-purin-6-amine (*m/z* 164.0935 [M + H]^+^). The tryptophan precursor metabolite, indirubin (*m/z* 263.0820 [M + H]^+^)[31], was putatively identified, further highlighting the diversity of alkaloids present in the bioactive extracts.

The analysis also yielded annotations for the phenylethylamide (PEAs) class of compounds (Fig. 6). This cluster comprised three nodes. Two nodes exhibited similar masses (isotopes) and were identified as phenylethylamide 357 (*m/z* 358.3109 [M + H]^+^), a compound previously characterized in the GNPS spectral library (https://gnps.ucsd.edu). The third node differed by 14 amu (*m/z* 372.3262 [M + H]^+^), consistent with typical CH_2_ losses in alkane straight chains, hence these compounds are appropriately classified as fatty acyls.

**Fig. 7:**
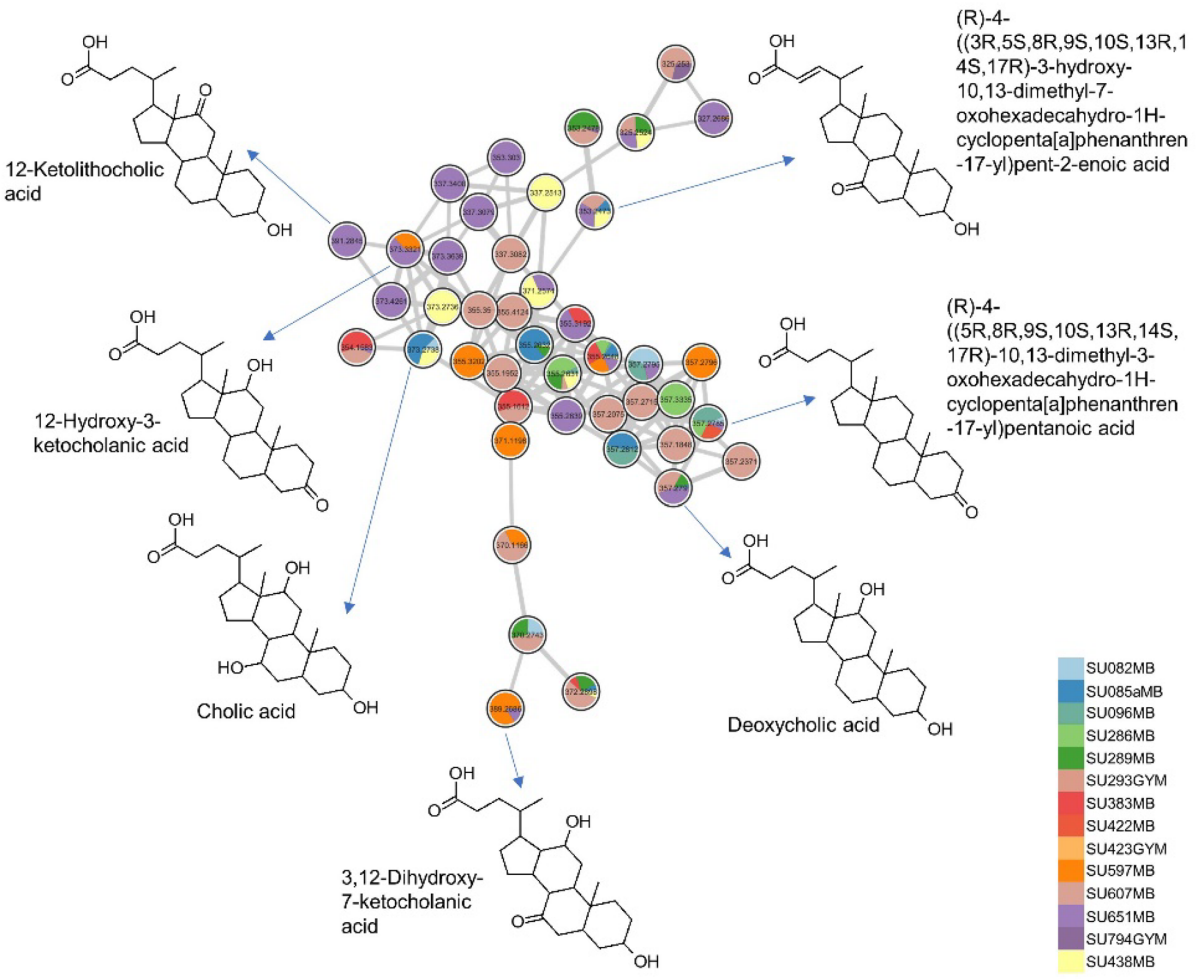
Putative bile acid cluster derived from 14 bacterial extracts

In the negative ion mode (Fig. S5), phosphatidylglycerols and phosphatidylethanolamines were mostly annotated. Additionally, sulfonolipids, such as sulfobacin B, was putatively identified from the extract of *Pseudoalteromonas* sp. SU088 that derived from the gut of the dragonfish *S. boa ferox.* Many nodes remain unannotated and this underscores the chemical complexity of the analyzed extracts and suggests potential to obtain new and bioactive molecules. The bile acid cluster was primarily composed of ions derived from 14 isolates mostly cultivated in MB medium (Fig. 7 and Fig. S4). Identified bile acid derivatives included cholic acid (*m/z* 373.2738 [M – 2H_2_O + H]^+^), deoxycholic acid (*m/z* 357.2790 [M-2H_2_O + H]^+^), (*R*)-4-((5*R*,8*R*,9*S*,10*S*,13*R*,14*S*,17*R*)-10,13-dimethyl-3-oxohexadecahydro-1H-cyclopenta[a]phenanthren-17-yl)pentanoic acid (*m/z* 357.2790 [M − H_2_O + H]^+^), 12-hydroxy-3-ketocholanic acid (*m/z* 373.2738 [M − H_2_O + H]^+^), 12-ketolithocholic acid (*m/z* 391.2845 [M + H]^+^), dihydroxy-7-ketocholanic acid (*m/z* 389.2686 [M − H_2_O + H]^+^) and (*R*)-4-((3*R*,5*S*,8*R*,9*S*,10*S*,13*R*,14*S*,17*R*)-3-hydroxy-10,13-dimethyl-7-oxohexadecahydro-1*H*-cyclopenta[a]phenanthren-17-yl)pent-2-enoic acid (*m/z* 353.2473 [M − 2H_2_O + H]^+^). Other bile acid conjugates annotated include 7-oxoglycochenodeoxycholic acid (*m/z* 470.2880 [M + Na]^+^) and methyl 3,7,12-trioxocholan-24-oate (*m/z* 439.2458 [M + Na]^+^). A list of all annotations can be found in Table S5. Bile acids are known to exhibit selective inhibitory activity against human pathogenic bacteria, including *Staphylococcus aureus*, *Pseudomonas aeruginosa*, and *Enterobacter cloacae* [32].

## Discussion

The microbiota associated with mesopelagic fauna represents a rich, underexplored resource for bioprospecting, offering potential for novel bioactive compounds with applications in pharmaceuticals and agriculture. In this study, 643 bacterial isolates were obtained from various mesopelagic animals, dominated by the phyla Pseudomonadota, Bacteroidota, and Actinomycetota, aligning with previous studies of microbial communities in mesopelagic zones [22, 33–35]. Notable genera such as Gram-negative *Sulfitobacter* and *Marinobacter* were present in multiple mesopelagic species, emphasizing their adaptability to different microenvironment. The actinobacterial *Dietzia sp.*, isolated from seven species, has potential applications in biodegradation, biosurfactant production, and biocolouring [36], while the Gram-positive *Arthrobacter spp.*, previously identified as a probiotic for shrimp post-larvae [37], may also play a similar role for fish larvae in the mesopelagic zone. Additionally, symbiotic bacteria such as *Photobacterium*, *Vibrio*, *Aliivibrio*, and *Shewanella* spp. assist in biomass breakdown and digestion in fish gut [38, 39]. Notably, 27 host-specific bacterial isolates highlight the ecological heterogeneity among study specimens, supporting the concept of microbiomes shaped by host physiology, diet, or environment [40]. Due to damage incurred during deep-sea trawling, skin samples are underrepresented in our dataset, limiting the full assessment of skin-associated microbial diversity. Considering that fish skin acts as a primary barrier and harbors distinct microbiota with antimicrobial potential [12], future studies should include targeted, intact skin sampling to capture this important niche. Despite the limitations of a culture-dependent microbial community study, our findings align with studies using Next-Generation Sequencing (NGS) which also found high variability and unique bacterial taxa in mesopelagic fish influenced by factors such as habitat preferences and behavior [22]. Our broad sampling strategy provided a wider spectrum of cultivable microbial diversity from the same marine environment. Notably, no fungal isolates were retrieved. This could be attributed to several factors, including the inherently low fungal abundance in mesopelagic environments, which are cold, nutrient-poor, and typically dominated by bacteria [41]. Herein we used only one medium (Wickerham, WSP) to obtain fungi. Other media (e.g., Potato Dextrose Agar, Saubouroud Agar) that were used in retrieval of psychrophilic fungi [42] could potentially have assisted the isolation of fungi. Although cultivation was performed at a cold temperature (at 6 °C) to mimic in situ temperatures, some deep-sea fungi may remain unculturable or require highly specific growth conditions not replicated in standard laboratory conditions. Molecular approaches, such as high throughput ITS amplicon sequencing may be necessary to detect such cryptic fungal communities [43]. Antibiotic discovery efforts have traditionally focused on Gram-positive bacteria. However, Gram-negative bacteria are being recognized as a rich, underexplored source of novel antimicrobials with potential to combat antibiotic resistance [44]. In this study, the biotechnological potential of the cultivable microbial communities associated to mesopelagic animals, dominated by Gram-negative bacteria, was evaluated. Extracts such as Gram-negative bacteria *Pseudoalteromonas* sp. SU088GYM and SU286GYM and actinobacteria *Agrococcus* sp. SU436GYM and *Paeniglutamicibacter* sp. SU836GYM, displayed potent activities against several pathogens. The majority of the microbial extracts exhibited significant inhibition against the ESKAPE pathogens *E. faecium*, MRSA, and the fish pathogen *L. garvieae*. Notably, *E. faecium* was the most susceptible pathogen, followed by *L. garvieae,* with several extracts showing moderate to high IC_50_ values, underscoring the promising antimicrobial potency of these isolates. The pronounced sensitivity of *L. garvieae*, a major re-emerging pathogen causing lactococcosis in fish and severely impacting aquaculture sustainability [45], to our extracts underscores the potential of the mesopelagic-associated bacteria as valuable sources of novel antibiotics to combat multidrug-resistant human pathogens and improving fish health in aquaculture. The limited anticancer activity observed may be attributed to the very crude nature of the extracts, where potentially active compounds are present at low concentrations or obscured by the other, more abundant constituents of the extracts. Additionally, the selected cancer cell lines may be insensitive to the bioactive metabolites present. Crude extract fractionation, compound purification, the use of a broader panel of cancer cell lines and higher test concentrations may provide further insights into the anticancer potential of the microbial extracts.

The 26 most bioactive extracts were subjected to untargeted metabolomics employing feature-based molecular networking (FBMN) to cluster features into chemical family clusters and dereplication tools such as SIRIUS + CANOPUS to predict their molecular formulae and chemical classes [23, 25]. Annotations were validated and extended by manual dereplication using databases such as Dictionary of Natural Products, MarinLit, SciFinder, Natural Product Atlas and COCONUT. This revealed a diverse array of predicted compound classes across multiple biosynthetic pathways, including alkaloids, terpenes, steroids, polyketides and lipids. This comprehensive metabolomic profile highlights the chemical diversity of the bioactive extracts, and also explains their observed bioactivity. For example, fatty acid compounds such as phosphoethanolomines, ornithine and lysine lipids were commonly annotated in the bacterial extracts. Chemical class annotations (CANOPUS) also identified the presence of unsaturated fatty acids, monoacylglycerols, diacylglycerols, and terpenoid classes, including steroids. These compounds are integral to the formation of the lipid bilayer membranes in bacterial cells [46–48]. Beyond their primary roles, the lipids exhibit significant antibacterial and biosurfactant properties [46], potentially contributing to the bioactivities observed in the current study. Phenylethylamides (PEAs) are bacterial metabolites that have been shown to competitively inhibit Se-5HTR receptors in insects, resulting in significant immunosuppression [49]. PEAs from fish-associated bacteria may open new avenues for pest control strategies and fish immune regulation. As displayed by CANOPUS, many different classes of compounds were predicted but not annotated to known compounds. These represent putative novel antimicrobial or otherwise active compounds with bioprospecting potential.

Although bile acids, steroidal class of compounds, were largely annotated in the Gammaproteobacteria *Psychrobacter* and *Pseudoalteromonas* extracts, some nodes in this cluster were derived from other species. This observation is consistent with previous studies that identified bile salt MS/MS (MS^2^) spectrum in Actinomycetia, Bacilli and Gammaproteobacteria, which are abundant in gut microbiota [50, 51]. Bile acids serve as physiological detergents that facilitate lipid metabolism and regulate energy metabolism. They are currently being investigated in clinical trials for the treatment of multiple sclerosis, inflammatory bowel disease, carcinoma, type 2 diabetes, gastroesophageal reflux disease, sepsis in malnourished children, peroxisomal disorders, asthma, and alcoholic hepatitis [51]. A recent study demonstrated that exogenous bile acids can maintain lipid and glucose homeostasis and also improve fish health and growth performance [52]. The presence of these bile acid-producing bacteria in the fish gut and gills suggests their involvement in maintaining the health and growth of fish. Additionally, bile acids possess antibiofilm and antimicrobial properties [32, 53, 54], which may contribute to observed bioactivity profiles of the active isolates in the current study.

Diketopiperazines and indole alkaloids are commonly produced by bacteria and have received significant attention due to their antimicrobial antitumor, antiinflammatory, antimalarial and antihyperglycemic properties [29, 55]. We annotated the diketopiperazines cyclo(L-Pro-L-Leu) and cyclo(L-Tyr-L-Pro), which displayed antifungal activity against *Aspergillus fumigatus* [56]. The alkaloid 5-hydroxyindole, derived from gut bacteria of human, has been identified as a potent stimulant of intestinal motility via its action on L-type calcium channels [57]. These compounds and their unidentified derivatives possess diverse pharmacological activities [58] and may contribute to the bioactivities exhibited by the isolates in this study.

Few studies have explored the bioactivities of mesopelagic microbiota. We recently identified aminolipids and bile salts from bacteria associated with the mesopelagic helmet jellyfish *Periphylla periphylla* [34]. A bacterial isolate, BTSS-3, from the gut of the deep-sea shark *Centroscyllium fabricii*, exhibited antimicrobial activity against several human pathogens, including *Salmonella typhimurium, Proteus vulgaris,* and *Clostridium perfringens* [59]. Hirano et al. [60] also reported chitinolytic activity of bacteria isolated from the mesopelagic copepod *Cephalophanes reflugens*, showing *Vibrio*, *Photobacterium*, and *Pseudoalteromonas* spp. to degrade colloidal chitin and shrimp carapace but no chemical/metabolome study was undertaken.

## Conclusions

The cultivable bacterial community associated with the mesopelagic specimens was dominated by Gram-negative Gammaproteobacteria. Our results suggest their relevance for the discovery of antimicrobial natural products for the pharmaceutical and aquacultural sectors. Our future studies will prioritize extracts, such as *Paeniglutamicibacter* sp. SU794GYM and *Pseudoalteromonas* sp. SU088GYM, which demonstrated high and broader activities plus more extract-specific unannotated clusters, for large scale cultivation and purification of bioactive compounds.

## Experimental section

### Sampling

Mesopelagic fish specimens (13 different fish species) and water were sampled from the North Atlantic Ocean, during the International Ecosystem Summer Survey in the Nordic Seas (IESSNS) in July 2020. The research expedition was conducted with the Icelandic research vessel "Árni Friðriksson." Collection was facilitated by the utilization of microzooplankton and Multpelt 832 pelagic trawls, strategically deployed at various stations (Fig. S1), with net deployment down to 1000 m. Samples included animals such as lanternfish, pearlside, dragonfish, as well as jellyfish, squid, krill and ambient seawater samples from some stations (Table 1). *Periphylla periphylla* was omitted from this study because we recently reported on diversity and biotechnological potential of its microbiota [34].

## Isolation of bacteria associated with mesopelagic animals

Isolation of microbes associated with the sampled species and seawater was performed using three different solid isolation media. These include Hastings medium (HS; Na_2_HPO_4_ * 12H_2_O: 9.35 g/L, KH_2_PO_4_: 1.00 g/L, (NH_4_)_2_SO_4_: 0.5 g/L, MgSO_4_* 7 H_2_O: 0.21 g/L, NaCl: 30 g/L, tryptone: 5 g/L, yeast extract: 3 g/L, glycerol: 2 mL/L), modified Wickerham medium (WSP; glucose monohydrate: 10 g/L, peptone from soymeal:5 g/L, malt extract: 3 g/L, yeast extract: 3 g/L, sodium chloride: 5 g/L) and marine broth medium (MB; Difco™ Marine Broth 2216: 37.4 g/L, pH 7.6 ± 0.2) [34]. Gills and gut tissues of fish samples were carefully and aseptically obtained and plated onto cultivation media as described previously by Ghotbi, et al. [12] with slight modifications. Briefly, the fish was aseptically dissected following rinsing in sterile saline. The gill and entire gut after the stomach were separately homogenized in microcentrifuge tubes (2 mL, Eppendorf, Germany) containing 500 µL of sterile saline by a sterile pestle and vortex. Unlike all other mesopelagic fish obtained during this cruise, the skin of fish *Xenodermichthys copei* and *Bathylagus euryops* remained intact and was swabbed by a sterile cotton swab and placed in 500 µL sterile seawater and mixed for 10 s. Swabs from the outer umbrella and surface tissues from the inner umbrella of the jellyfish *Atolla* sp. were processed as described in previous work [34]. For the squid sample, surface swabs, intestines and ink sac (shiny appearance) were aseptically obtained and separately homogenized. Sterile surface swabs were taken for the krill sample. Homogenized samples were mixed for 10 s and dilutions of 10^–2^ in sterile saline were prepared and plated (100 µL) onto the isolation media. Water samples (100 µL) were simply plated onto isolation media (Table S1). Plates were incubated at 6 °C to simulate natural temperature. Colonies were selectively picked from the plates at intervals spanning 2 to 28 days of incubation. These colonies were then transferred to fresh agar plates until pure cultures were obtained. Cryopreservation of all strains was performed at -80 °C utilizing the Microbank™ system (Pro-Lab Diagnostics, Richmond Hill, ON, Canada).

## Identification of bacteria associated mesopelagic animals

Identification of isolates was conducted by molecular methods. Genomic DNA extraction was carried out using either a freeze–thaw protocol [61] or the DNeasy Plant Mini Kit (Qiagen, Hilden, Germany) following manufacturer’s guidelines. PCR amplification of the 16S rRNA gene was accomplished using universal primers Eub27f and 1387r and a cycling protocol previously described by Utermann et al. [62]. Amplified DNA fragments of the correct length underwent Sanger sequencing at LGC Genomics GmbH (Berlin, Germany). Resulting sequences were subjected to quality checks and trimming, formatted in FASTA format and subjected to BLAST analysis against the NCBI nucleotide database. The bacterial 16S rRNA gene sequences were deposited in GenBank with accession numbers PQ176822—PQ177463 (Table S2).

## Cultivation and extraction of bacteria associated with mesopelagic animals

A careful selection of the microbial strains was performed. This was based on (1) biological safety (exclusion of BSL-2 organisms), (2) sampling tissue of the mesopelagic animal, (3) sampling location, and (4) phylogeny of the isolates to exclude identical strains from the same source and also to reflect a broad diversity. This resulted in a total of 394 bacterial isolates for cultivation. Selected isolates were cultured in two liquid growth media: a low-nutrient Marine broth (MB) and a high-nutrient Glucose Yeast Malt (GYM; D-glucose monohydrate: 20 g/L, malt extract: 4 g/L, yeast extract: 4 g/L, calcium carbonate: 2 g/L) medium. Bacterial cultures were maintained in MB at 22 °C (with shaking at 120 rpm) for 7 days as pre-cultures. Main cultures were inoculated with an optical density (OD_600_) of 0.01 in 500 mL medium (2 L Erlenmayer flasks). Cultures were subsequently incubated for 7 days with shaking at 120 rpm in the dark. Some bacterial isolates did not grow in the selected media. Grown cultures were extracted with EtOAc as previously described for liquid cultures [34]. Media blanks (MB and GYM) were also extracted as controls. Aliquots (1 mg) of all extracts were transferred into separate vials for bioactivity testing and UPLC-MS/MS profiling.

## Antimicrobial assays

All crude extracts were screened against the human nosocomial pathogens, ESKAPE panel, comprising *Enterococcus faecium* (Efm, DSM 20477), methicillin-resistant *Staphylococcus aureus* (MRSA, DSM 18827), *Klebsiella pneumoniae* (Kp, DSM 30104), *Acinetobacter baumannii* (Ab, DSM 30007), *Pseudomonas aeruginosa* (Psa, DSM 1128) and *Escherichia coli* (Ec, DSM 1576). Additionally, screenings were conducted against human pathogenic yeasts *Candida albicans* (Ca, DSM 1386) and *Cryptococcus neoformans* (Cn, CDSM 6973) as well as the fish pathogenic bacteria *Lactococcus garvieae* (Lg, DSM 20684) and *Vibrio ichthyoenteri* (Vi, DSM 14397). Test pathogens were purchased from Leibniz Institute DSMZ-German Collection of Microorganisms and Cell Cultures (Braunschweig, Germany). The assays were carried out by the broth dilution approach in 96-well microtiter plates following established protocols as described in previous work [34]. Bioactivity testing was conducted at an effective test concentration of 100 µg/mL from crude extracts prepared at a concentration of 20 mg/mL in dimethyl sulfoxide (DMSO, Carl Roth). Growth media (pathogen specific, Table S3) and 0.5% DMSO were tested as negative controls. Positive controls included amphotericin (Cn), ampicillin (Efm, Lg), chloramphenicol (MRSA, Ec, Kp, Vi), doxycycline (Ab), nystatin (Ca) and polymyxin B (Psa). IC_50_ values were further calculated for extracts that exhibited over 80% inhibition, at 100 µg/mL, against three or more pathogens.

## Anticancer assays

Crude extracts were also assessed against two human cancer cell lines, i.e., melanoma cell line A-375 and colon cancer cell line HCT-116. To assess general toxicity, the human keratinocyte cell line HaCaT was included. All cell lines were procured from CLS Cell Lines Service (Eppelheim, Germany). The anticancer activity of the extracts was assessed by monitoring the metabolic activity of the cell lines using the CellTiterBlue Cell Viability Assay (Promega, Mannheim, Germany) as outlined in previous work [63]. Testing was conducted at an effective test concentration of 100 µg/mL. Doxorubicin was used as positive control, 0.5% DMSO and growth media were used as negative controls.

## UPLC-MSMS analyses

Analysis was performed on an Acquity UPLC I-Class System (Waters®, Milford, MA, USA) coupled to a Xevo G2-XS quadrupole-time-of-flight mass spectrometer with an electrospray ionization source (Waters^®^, Milford, MA, USA). Extracts were dissolved in MeOH at a final concentration of 1 mg/mL and injected (0.3 µL) onto an Acquity HSS T3 C18 column (High Strength Silica C18, 1.8 mm, 100 × 2.1 mm, Waters) at a temperature of 40 °C. The mobile phase consisted of a mixture of (A) water with 0.1% formic acid, and (B) acetonitrile with 0.1% formic acid at a pumping rate of 0.4 mL/min. Analyses started at 5% B for 2 min, followed by a linear gradient of 5–100% B over 12 min and isocratic at 100% B for 3 min, back to initial condition in 1 min and concluded with a reconditioning phase until minute 20. MS was performed in fast DDA acquisition mode, with an ESI source over a mass range of *m/z* 50–1200 Da in both positive and negative ion modes, with a capillary voltage held at 2.2 kV, cone gas flow of 50 L/h, desolvation gas flow of 1000 L/h, source temperature of 150 °C, and desolvation temperature of 500 °C, with the sampling cone and source offset at 40 and 50, respectively. The data dependent MS/MS events were performed on the 5 most intense ions in full scan MS. The MS/MS isolation window width was 2 Da, and the collision energy was ramp with the following settings: LM CE ramp start = 10, LM CE ramp end = 40, HM CE ramp start = 10, HM CE ramp end = 40, and a scan rate of 0.25 s in centroid data format. After acquisition in a MS/MS scan, parent ions were placed in a dynamic exclusion list for 3.0 s. All measurements were performed in triplicate and mass-corrected with LockSpray (reference masses: 120.0813 and 556.2771 Da MSMS in positive ion mode and 554.2615 Da MSMS in negative ion mode for Leucine enkephalin). Data were analyzed with MassLynx® software (v4.2).

## Molecular networking and compound annotations

The MS^2^ data were converted from the .RAW (Waters) data format to .mzXML format using ProteoWizard msconvert (version 3.2.20033; Vanderbilt University, Nashville, TN, USA) [64] and then processed with MZmine 3 [65]. SIRIUS output file.mgf, was used with Sirius + CSI:Finger-ID and CANOPUS [24] to obtain molecular formula predictions, structural annotations and chemical class assignments. MZmine output files, in both .csv and .mgf, formats, were exported to the Global Natural Products Social Molecular Networking (GNPS, https://gnps.ucsd.edu) platform, where networks were generated using the feature-based molecular networking (FBMN) workflow [23, 28]. Parameters used were adopted from previous work [67]. Edges were filtered to include only those with a cosine score > 0.75. Network spectral hits to library spectra were required to have a score > 0.7. Cytoscape was used to visually display the data as a network of nodes (consensus spectra) and edges (connection between nodes) [66]. Nodes originating from control samples (MeOH and cultivation media) were excluded.

Additionally, compound annotations were extended by predicting the molecular formula of parent ions using MassLynx version 4.2, and subsequently searching them against various databases, including Dictionary of Natural Products (https://dnp.chemnetbase.com, accessed on 10 December 2024), MarinLit (https://marinlit.rsc.org/, accessed on 10 December 2024), SciFinder (https://scifinder-n.cas.org, accessed on 10 December 2024), The Natural Product Atlas (https://www.npatlas.org/, accessed on 10 December 2024), and COCONUT (https://coconut.naturalproducts.net/, accessed on 15 December 2024).

## Supplementary Information

Additional file 1. Fig. S1. Sampling locations along the transect of the IESSNS cruise. Fig S2. Diversity of isolated bacteria at phylum level at different sampling points. Fig. S3. Distribution of fish isolates based on tissue sampled. Fig. S4. Molecular network derived from positive ion mode MS/MS data. Fig. S5. Molecular network derived from negative ion mode MS/MS data. Table S1. Mesopelagic species and tissue types used for microbial isolation. Table S3. Test pathogens, their cultivation media and positive controls for bioactivity testing.

Additional file 2. Table S2. Bacterial isolates. Table S4. Bioactivity profiling. Table S5. Metabolite annotation.

## Data Availability

The MS datasets generated and analyzed for this study can be found in the MassIVE Public GNPS database [MSV000095612]. The molecular networking jobs can be publicly accessed at https://gnps2.org/status?task=7b0589560b464771ac8e6c9d6aaf04d5 (positive ion mode) and https://gnps2.org/status?task=1a7437433f334dc5a0169bcb6d73f49f (negative ion mode). 16S rRNA gene sequences of the mesopelagic bacteria can be found in the Genbank [PQ176822—PQ177463]. All relevant data are within the article and its supplementary information.
