# Supplementary material for: Bioprospecting cultivable bacteria associated with deep sea (mesopelagic) fish of the North Atlantic Ocean
Source: Nat Prod Bioprospect. 2025 Jul 2;15(1):42. doi: 10.1007/s13659-025-00527-6 (PMC12222571; doi:10.1007/s13659-025-00527-6)
Supplement: Supplementary file 1 — Additional file 1. Fig. S1. Sampling locations along the transect of the IESSNS cruise. Fig S2. Diversity of isolated bacteria at phylum level at different sampling points. Fig. S3. Distribution of fish isolates based on tissue sampled. Fig. S4. Molecular network derived from positive ion mode MS/MS data. Fig. S5. Molecular network derived from negative ion mode MS/MS data. Table S1. Mesopelagic species and tissue types used for microbial isolation. Table S3. Test pathogens, their cultivation media and positive controls for bioactivity testing. [file 13659_2025_527_MOESM1_ESM.docx]

Supplementary Material

Bioprospecting Cultivable Bacteria Associated with Deep Sea (Mesopelagic) Fish of the North Atlantic Ocean

Ernest Oppong-Danquah^1^, Jana Heumann^1^, Hannah Moosbauer^1^, Martina Blümel^1^, Arlette Wenzel-Storjohann^1^, and Deniz Tasdemir^1,2*^

^1^GEOMAR Centre for Marine Biotechnology (GEOMAR-Biotech), Research Unit Marine Natural Products Chemistry, GEOMAR Helmholtz Centre for Ocean Research Kiel, Kiel, Germany

^2^Kiel University, Kiel, Germany

*** Correspondence:**Deniz Tasdemir
[dtasdemir@geomar.de](mailto:dtasdemir@geomar.de)

**List of Supplementary Figures Page No.**

**Fig. S1.** Sampling locations along the transect of the IESSNS cruise 2

**Fig. S 2.** Diversity of isolated bacteria at phylum level at different sampling points 2

**Fig. S 3.** Distribution of fish isolates based on tissue sampled 3

**Fig. S 4.** Molecular network derived from positive ion mode MS/MS data 4

**Fig. S 5.** Molecular network derived from negative ion mode MS/MS data 5

**List of Supplementary Tables Page No.**

**Table S1**. Mesopelagic species and tissue types used for microbial isolation 6

**Table S3.** Test pathogens, their cultivation media and positive controls for bioactivity testing 7

**Tables S2**, **S4** and **S5** are on supplementary excel sheet


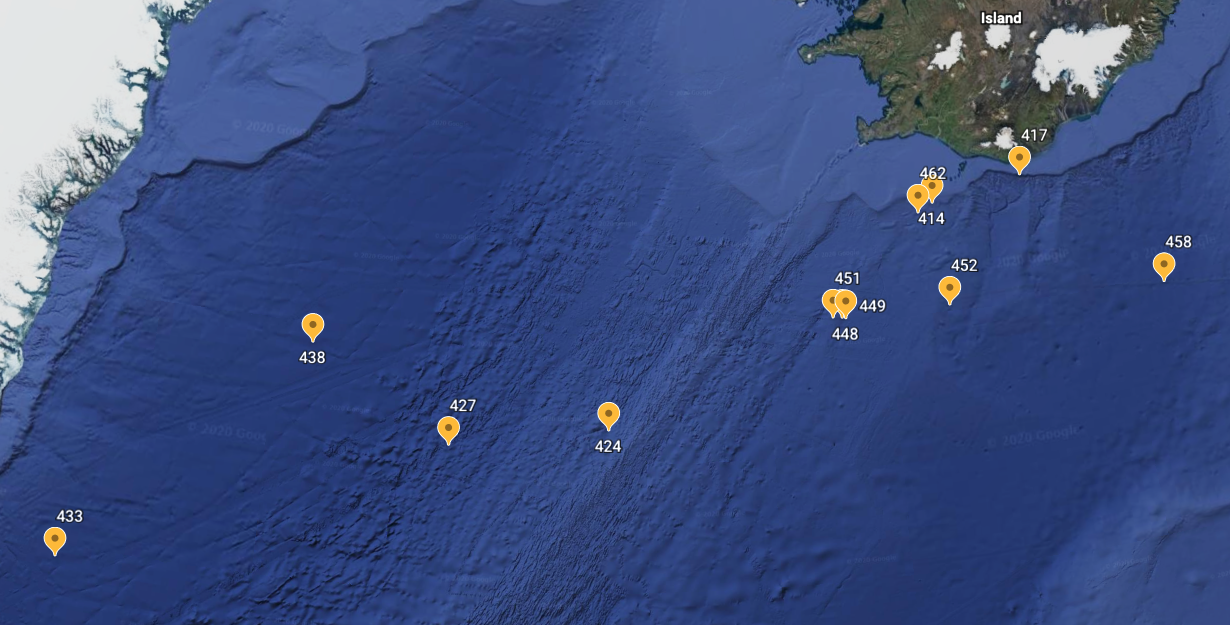


Cruise track IESSNS 13.-30.7.20

Irminger Sea

Greenland

Iceland


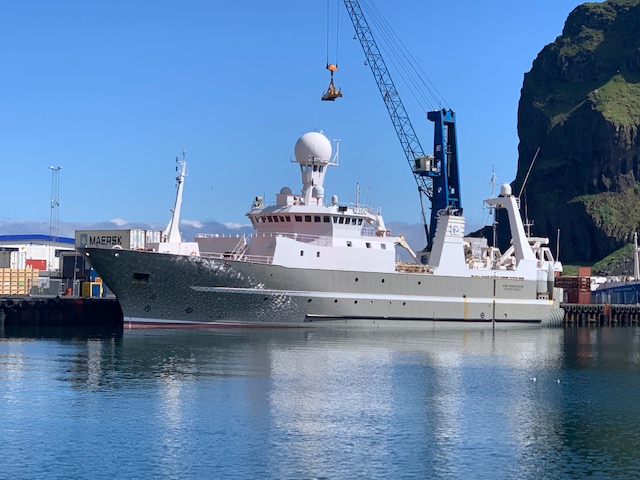


**Fig. S1** Sampling locations along the transect of the International Ecosystem Summer Survey in the Nordic Seas (IESSNS) cruise.

**Fig. S2** Diversity of isolated bacteria at phylum level from mesopelagic animals at different sampling stations. Geographical coordinate for all sampling stations are provided in Table 1.

**Fig. S3** Distribution of fish isolates based on tissue sampled.


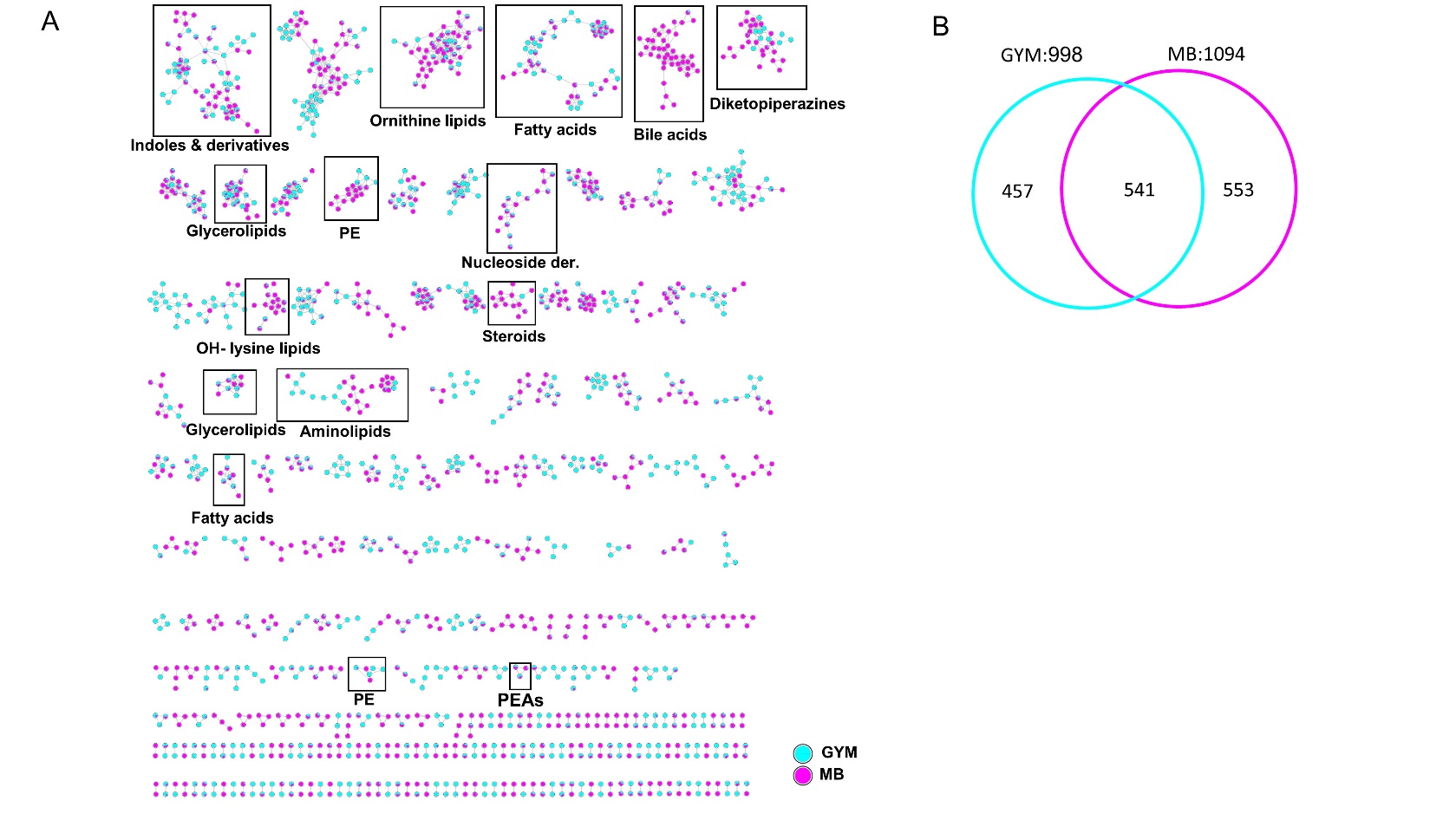


**Fig. S4** (A) Molecular network derived from positive ion MS/MS data of the 26 most active extracts (B) Euler diagram displaying the peak ion distribution among the culture media Marine Broth (MB) and Glucose-Yeast-Malt (GYM). PE: phosphatidylethanolamines, PEAs: phenylethylamides.


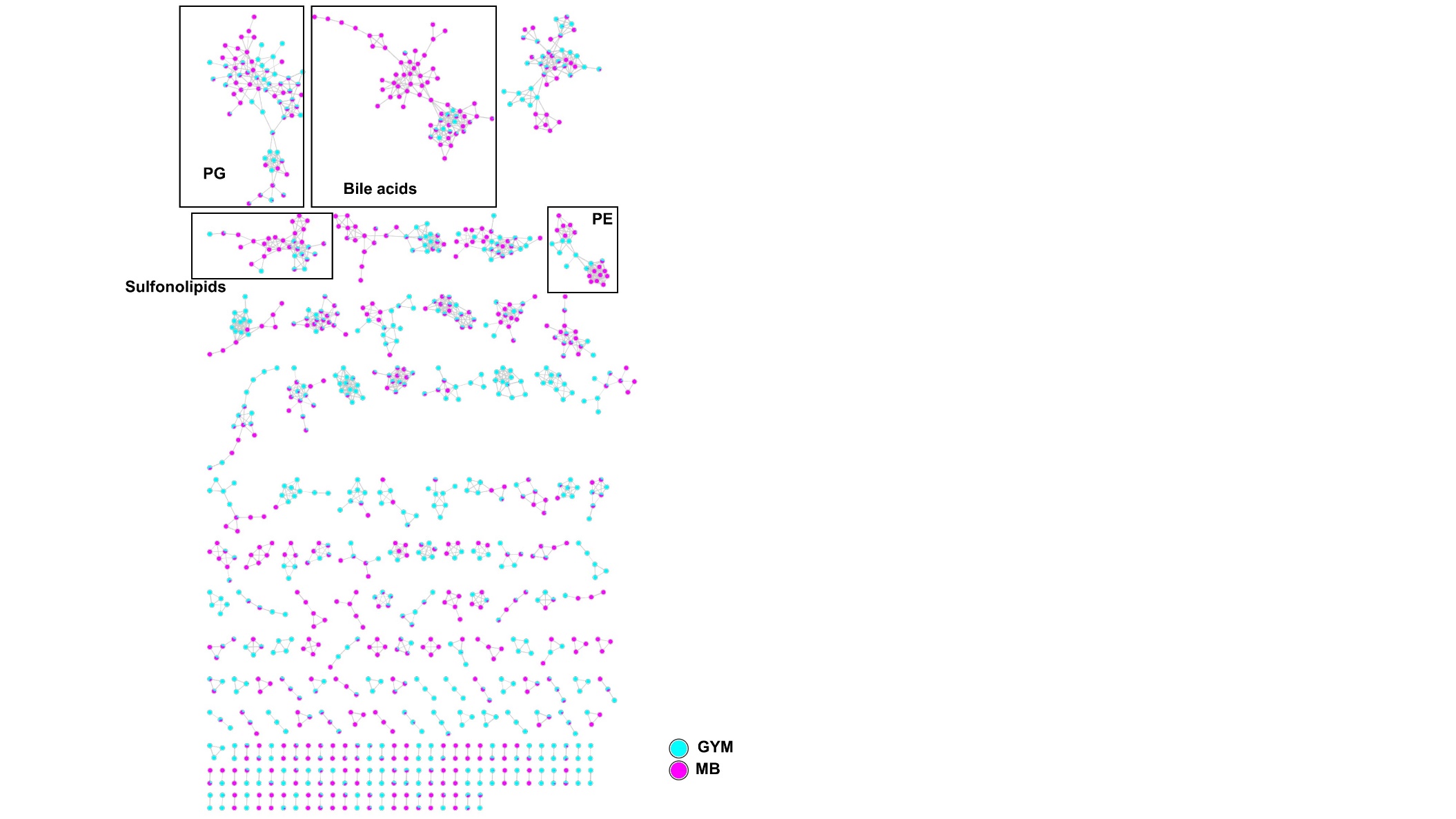


**Fig. S5** Molecular network derived from negative ion mode MS/MS data of the 26 most active extracts according to cultivation media Marine Broth (MB) and GYM (Glucose-Yeast-Malt). PE: phosphatidylethanolamines, PG: phosphatidylglycerol.

**Table S1** Mesopelagic species and tissue types used for microbial isolation

| Sample type | Species | Family | Tissue | Amount plated |
| --- | --- | --- | --- | --- |
| Fish | *Arctozenus rissoi* | Paralepididae | Gill in 500 µl sterile seawater Gut after stomach | 1:100 dilution plated |
| Fish | *Bathylagus euryops* | Bathylagidae | Skin swab, 1:100 dilution plated Gill in 500 µl sterile seawater Gut in 500 µl sterile seawater | 1:100 dilution plated |
| Fish | *Benthosema glaciale* | Myctophidae | Gill in 500 µl sterile seawater Gut in 500 µl sterile seawater | 1:100 dilution plated |
| Fish | *Cyclothone microdon* | Gonostomatidae | Gill in 500 µl sterile seawater Gut in 500 µl sterile seawater | 1:100 dilution plated |
| Fish | *Chauliodus sloani* | Stomiidae | Gill in 500 µl sterile seawater Gut in 500 µl sterile seawater | 1:100 dilution plated |
| Fish | *Myctophum punctatum* | Myctophidae | Gill in 500 µl sterile seawater Gut in 500 µl sterile seawater | 1:100 dilution plated |
| Fish | *Maurolicus muelleri* | Sternoptychidae | Outer surface of umbrella Inner surface of umbrella | 1:100 dilution plated |
| Fish | *Notoscopelus kroyeri* | Myctophidae | Gill in 500 µl sterile seawater Gut after stomach | 1:100 dilution plated |
| Fish | *Protomyctophum arcticum* | Myctophidae | Outer surface of umbrella Inner surface of umbrella | 1:100 dilution plated |
| Fish | *Paralepis coregonoides* | Paralepididae | Outer surface of umbrella Inner surface of umbrella | 1:100 dilution plated |
| Fish | *Scopelogadus beanii* | Melamphaidae | Gill in 500 µl sterile seawater | 1:100 dilution plated |
| Fish | *Stomias boa ferox* | Stomiidae | Gill in 500 µl sterile seawater Gut in 500 µl sterile seawater | 1:100 dilution plated |
| Fish | *Xenodermichthys copei* | Alepocephalidae | Skin swab, 1:100 dilution plated Gill in 500 µl sterile seawater Gut in 500 µl sterile seawater | 1:100 dilution plated |
| Jellyfish | *Atolla* sp. | Atollidae | Surface swab Inner hat tissue of jellyfish | 1:100 dilution plated |
| Krill | *Meganyctiphanes norvegica* | Euphausiidae | Surface swab | 1:100 dilution plated |
| Squid | *Gonatus sp.* | Gonatidae | Surface swab Inner body, intestines  Inner body, ink sac | 1:100 dilution plated |
| Seawater | Seawater | Seawater | 100 µl plated | 100 µl plated |

**Table S3** Test pathogens, their cultivation media and positive controls for bioactivity testing

|  |  |  |  |
| --- | --- | --- | --- |
| **Test pathogen** | **Abbreviation** | **Positive control (conc.)** | **Medium** |
| *Escherichia coli* | Ec | 10µM Chloramphenicol | TSB12 |
| Methillicin resistent *Staphylococcus aureus* | MRSA | 10µM Chloramphenicol | TSB12 |
| *Klebsiella pneumoniae* | Kp | 10µM Chloramphenicol | TSB12 |
| *Acinetobacter baumannii* | Ab | 10µM Doxocycline | TSB12 |
| *Pseudomonas aeruginosa* | Psa | 10µM Polymyxin B | TSB12 |
| *Enterococcus faecium* | Efm | 10µM Ampicillin | M92 |
| *Candida albicans* | Ca | 10µM Nystatin | M186/3 |
| *Cryptococcus neoformans* | Cn | 10µM Amphotericin B | M186 |
| *Lactococcus garviae* | Lg | 10µM Ampicillin | M92 |
| *Vibrio ichthyoenteri* | Vi | 10µM Chloramphenicol | MB |
| Human melanoma | A-375 | 100µg/ml Doxorubicin | RPMI + 10/FBS + 1%PS |
| Colon cancer | HCT-116 | 100µg/ml Doxorubicin | DMEM + 10%FBS + 1%PS |
| Human keratinocytes | HaCaT | 100µg/ml Doxorubicin | DMEM + 10%FBS + 1%PS |

TSB12: 12g tryptic soy broth, 5g NaCl in 1L H_2_O

M92: 30g tryptic soy broth, 3g yeast extract in 1L H_2_O (pH 7,0-7,2)
M186: 3g yeast extract, 3g malt extract, 5g peptone from soybeans, 10g Glucose
M186/3: M186 1:3 diluted

DMEM: (Dulbecco's Modified Eagle Medium)

FBS: Fetal bovine serum

PS: Sodium Pyruvate
